# The Lake Chad hydrology under current climate change

**DOI:** 10.1038/s41598-020-62417-w

**Published:** 2020-03-26

**Authors:** Binh Pham-Duc, Florence Sylvestre, Fabrice Papa, Frédéric Frappart, Camille Bouchez, Jean-Francois Crétaux

**Affiliations:** 1 Aix-Marseille Université, CNRS, IRD, Collège de France, INRAE, CEREGE, Europôle de l’Arbois, Aix-en-Provence, France; 2 Department of Space and Applications, University of Science and Technology of Hanoi, Vietnam Academy of Science and Technology, Hanoi, Vietnam; 3 LEGOS, Université de Toulouse, IRD, CNES, CNRS, UPS, Toulouse, France; 4 Universidade de Brasília, Institute of Geosciences, Campus Universitario Darcy Ribeiro, 70910-900 Brasilia, DF Brazil; 5 Université Rennes, CNRS, Géosciences Rennes, UMR 6118 Rennes, France

**Keywords:** Environmental sciences, Hydrology

## Abstract

Lake Chad, in the Sahelian zone of west-central Africa, provides food and water to ~50 million people and supports unique ecosystems and biodiversity. In the past decades, it became a symbol of current climate change, held up by its dramatic shrinkage in the 1980s. Despites a partial recovery in response to increased Sahelian precipitation in the 1990s, Lake Chad is still facing major threats and its contemporary variability under climate change remains highly uncertain. Here, using a new multi-satellite approach, we show that Lake Chad extent has remained stable during the last two decades, despite a slight decrease of its northern pool. Moreover, since the 2000s, groundwater, which contributes to ~70% of Lake Chad’s annual water storage change, is increasing due to water supply provided by its two main tributaries. Our results indicate that in tandem with groundwater and tropical origin of water supply, over the last two decades, Lake Chad is not shrinking and recovers seasonally its surface water extent and volume. This study provides a robust regional understanding of current hydrology and changes in the Lake Chad region, giving a basis for developing future climate adaptation strategies.

## Introduction

Severe and recurrent droughts are the principal weather-related hazards for developing economies throughout sub-Saharan Africa, and the quality of long-term weather prediction is a bottleneck hampering drought mitigation and adaptation in the region. This is aggravated by uncertain impacts of the 21^*st*^ century anthropogenic climate change on the continent’s rainfall and freshwater resources, due to at best fragmentary understanding on the effects of a warming atmosphere on the hydrological cycle at regional scales^1^. After severe and prolonged droughts that affected the Sahel in the 1970s–1980s which were considered as one of the first major consequences of global climate change during the 20^*th*^ century^2^, an increase in the mean annual precipitation was recorded since the beginning of the 1990s^3,4^. Despite of this trend, the current climate change seems to impact the Sahelian zone of west-central Africa with higher interannual variability^5^ affecting from year to year to the amount of precipitation during rainy seasons, and increasing the vulnerability of the regional economy, mainly based on agropastoral activities.

In this context, Lake Chad, located in the central Sahel sector, at the southern edge of the Sahara, rises up as a symbol of the current global climate change occurring in the region. After being ranked at the world’s sixth largest inland water body with an open water area of 25,000 km^2^ in the 1960s, it shrunk dramatically at the beginning of the 1970s and reduced to less than 2,000 km^2^ during the 1980s, decreasing by more than 90% its area^6^. The consequence of the 1970s and 1980s droughts was the subdivision of the lake into a northern pool and a southern pool, and the regular dryness of the northern pool alerted the international community of a possible lake’s disappearance^7^. It also illustrated the impact of extreme and rapid climate changes in this area as it was initially thought that water withdrawals for irrigation contributed to the lake decline^8^. Yet, recent studies showed that the amount of water extraction in the 1980s and 1990s was probably overestimated as the quantity of water abstracted for human activities was negligible compared to the lake volume change^9^.

Since the 1990s, it has been observed that the lake’s surface water extent has increased due to more favorable rainfall in western Sahel. Some studies estimated a peak of water extent in April 2013 with its maximum of 14,000 km^2^ ^10^. However, these estimates were usually based on partial observations and thus provided incomplete information and increased misunderstanding of the Lake Chad hydrological cycle. Other studies only investigated the southern pool of the lake^11^, during some months in a year^12^, or only focused on groundwater^13^. Only two studies investigated in details the seasonal variations of the Lake Chad, but focusing on the previous decades, the 1980s and the 1990s^6,14^.

Now, in the view of the challenges arisen in the Lake Chad area during the last decades, it is crucial to better characterize the Lake Chad hydrological cycle. Indeed, the Lake Chad region is currently facing multiple security risks, including livelihood and violent conflicts. Even though the current conflict was triggered by violence linked to the armed groups known as Boko Haram, the crisis has deep roots in longstanding challenges. Widespread inequality and decades of political marginalization have instilled and entrenched sense of exclusion in the region^15^. But several observations demonstrated that these challenges are further exacerbated by climate change^16^. Climate change is widely accepted to be a ’threat multiplier’ which exacerbates existing risks and worsen already fragile situations, making it harder to promote peace, adaptation and sustainable development^17^. In case of the Lake Chad region, the uncertainties of the unpredictable rainfall patterns induced by climate change are significantly impacting the resilience of the communities, and challenging the international community to promote an appropriate peaceful and sustainable development activities.

In this study, we quantify the variations of freshwater storage over the Lake Chad watershed and analyze the variability of the hydrological cycle over the region during the last two decades using multi-satellite obserations. Due to the decrease in number of hydrometeorological gauge stations and difficulties to obtain data consistently and accurately from remote regions, we combine complementary information from different satellite observations, e.g. optical imagery from MODIS and multi-missions satellite altimetry from Topex-Poseidon, Jason-1, 2, 3, ENVISAT and SARAL in order to reconstruct surface water extent and level of the Lake Chad (see Methods). This multi-sensor approach is particularly important because it allows to better evaluate the open water surface compared to the surface covered by vegetation, which is one of the main question when applying remote sensing techniques in the context of shallow lakes, especially the Lake Chad^14^. These complementary satellite-based products are combined to estimate the variation of surface water storage (SWS), which is then subtracted from the terrestrial water storage (TWS) derived from the Gravity Recovery and Climate Experiment (GRACE) satellite observations, for determining the relative contributions of soil moisture and groundwater variations to the Lake Chad basin (see Methods).

## Lake Chad - A complex hydrological system

**Figure 1:**
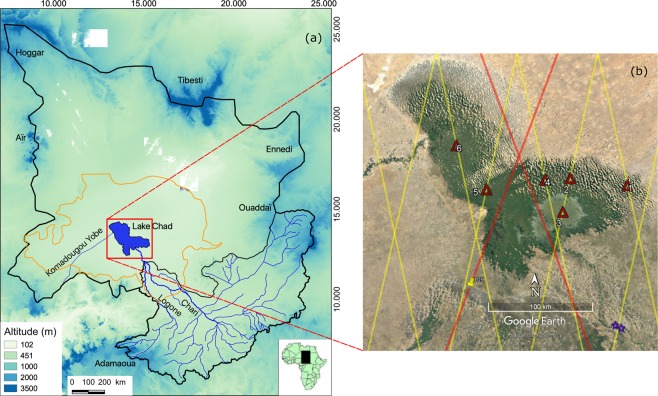
(**a**) Location map of the Lake Chad Basin (~2.5 × 10^6^ km^2^) with the delimitation of the conventional basin, the Quaternary aquifer (orange), and the Chari-Logone Basin (black). (**b**) The study area with ENVISAT/SARAL satellite altimeter ground-tracks (yellow), Topex-Poseidon/Jason ground-tracks (red), locations of six VSs (red triangles), and locations of N’Djamena and Chagoua gauge stations (blue stars). a was generated by QGIS Version 3.10.2^18^), and b was generated by Google Earth Pro Version 7.3 (https://www.google.com/earth/).

The Lake Chad drainage basin covers ~2.5 × 10^6^ km^2^, representing ~8% of the African continent (Fig. 1a). It is a hydrologically closed drainage system in the Central Sahel region of northern Africa, characterized by a south to north climatic gradient as a consequence of latitudinally decreasing rainfall. Runoff and river discharge are generated predominantly in the southern portion of the drainage basin and transported via the Chari/Logone river system to the lake which are >90%; the remaining coming from the Komadugu Yobe River and precipitation on the lake surface. These inputs are balanced by evaporation estimated at >2000 mm year^−1^ and seepage to groundwater^19^. Lake Chad is the terminal lake of this drainage system. Today, it is a shallow lake (<3 m), subdivided most of the time into three areas, the northern and the southern pools separated by an east-west vegetation-covered sand barrier named ‘The Great Barrier’^20^. The third one located at the eastern part of the lake, named ‘The Archipelagos’, corresponds to an inland area formed by sand dunes which are inundated according to the seasonally water inflow into the lake (Fig. 1b).

The lake is connected to an unconfined aquifer of 500,000 km^2^, the Quaternary Phreatic Aquifer (QPA)^21^. In present-day conditions, the aquifer is recharged by rainfall and seepage from the lake to the Quaternary Phreatic Aquifer. This reservoir ensures the chemical regulation of the lake and allows freshwater to persist despite the strong evaporative conditions^22^. This Quaternary Phreatic Aquifer is separated from the underlying sedimentary aquifers of the Continental Terminal and Pliocene by a thick clay layer^21^. These two continental sediment layers are separated aquifers in the Nigerian part of the basin and merge below Lake Chad to form a single aquifer with a total thickness exceeding 275 m. They are confined and of artesian type. Deep aquifers groundwater are mainly exploited in northern Nigeria and in eastern Niger.

## Land Surface Water Extent during the last two decades

**Figure 2:**
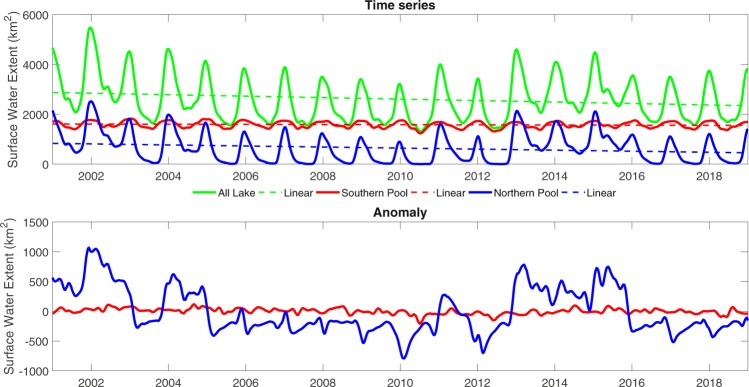
Time series (top) and anomaly (bottom) of surface water extent of the northern pool (blue), southern pool (red) and Lake Chad (green), for the 2001–2018 period. The trends are also plotted.

Times series and anomaly of the surface water extent of Lake Chad derived from multi-spectral MODIS images (see Methods/supporting document) show that the southern pool area is quite stable over the last two decades, with a nearly flat linear trend, meaning that its water surface current has not been affected by drastical changes since 2000s (Fig. 2). Examples of Lake Chad’s surface water extent maps derived from MODIS data are shown in Fig. S3 in the supporting information. Monthly average minimum and maximum surface water extent of the southern pool during this period are 1520 ± 60 km^2^ and 1830 ± 40 km^2^ in August and January, respectively. In the northern pool, surface water slightly decreases (Fig. 2). The northern pool shows higher variability, with completely dry periods during dry seasons between 2005 and 2012, whereas it was partly inundated during dry seasons in other periods between 2001 and 2004, and between 2013 and 2015. However, compared to the 1970s and 1980s decades when the northern pool was totally dry during both dry and wet seasons, until the beginning of the 2000s, water was still coming every year during rainy season with an amount variable from year to year. As the consequence, the total surface water extent of Lake Chad slightly reduced over the last 20 years, mostly due to the decreasing trend of the northern pool. The descending trend of surface water extent was higher during the 2001–2009 period compared to the 2010–2018 period. Lake Chad’s surface water extent was maximum in 2001–2002 (~5800 km^2^), then it continuously declined to its minimum in 2010 (~1800 km^2^) before starting to slowly increase again in the following years. Compared to previous studies, Lake Chad’s surface water extent are lower than what was already reported^6,12^ where the minimum, maximum and average of its surface water extent in dry seasons from 1988–1989 to 2016–2017 were estimated at 12,700 km^2^, 16,800 km^2^ and 6,400 km^2^, respectively. The large difference mainly comes from the way to define surface water. In this study, we only consider open water bodies as surface water, whereas, in previous studies, water under vegetation was included as surface water. If we assumed that all vegetation area of Lake Chad is also inundation, then the total surface water extent in October 2013 was 12,800 km^2^, in the range of previous results, e.g. 14,000 km^2^, reported in others studies^10,12^. Similarly, recent studies^11,13^ reported the minimum, maximum and average size of Lake Chad for the 2003–2016 period were only 1242 km^2^, 2231 km^2^ and 1694 km^2^, respectively. These numbers are 2–3 times lower than what we report here. But if we only consider the southern pool as it was done in previous studies, we found its monthly average minimum and maximum surface water extent are 1520 km^2^ and 1830 km^2^, which are close to what have been already reported^11^ (1427 km^2^ and 1465 km^2^, respectively).

The decrease of surface water extent in the northern pool can be explained by a combination of several factors. The decrease of the Komadugu Yobe discharge during the last decades could partly explain this decreasing trend^23^. Another driving factor could be the increase of permanent vegetation cover within Lake Chad. With the lowering of the lake during the 1970s and the 1980s combining with the increase of temperature, Lake Chad permanent vegetation cover increased by ~30% during the last two decades, from ~3800 km^2^ in the 2000s to ~5200 km^2^ today (Fig. S9 in supporting information). As a consequence, this increase of permanent vegetation cover could partly contribute to a higher evapotranspiration, which has been estimated at 200 mm year^−1^ higher on the northern pool than on the southern pool^19^. This increase of the permanent vegetation cover also contributes to the reduction of the runoff from the southern pool to the northern pool through the Great Barrier. Since the severe droughts during the 1980s, Lake Chad split into two pools, increasing favorable conditions for the vegetation growth, especially over the Great Barrier. For instance, it has been suggested by modeling experiments that without the split between the two pools during the 1980s, the northern pool should never dry out and should have recovered 82% of its 1963’s water area which was one of the highest extension of the lake during the last 60 years^24^.

## Surface and sub-surface water volume variations

**Figure 3:**
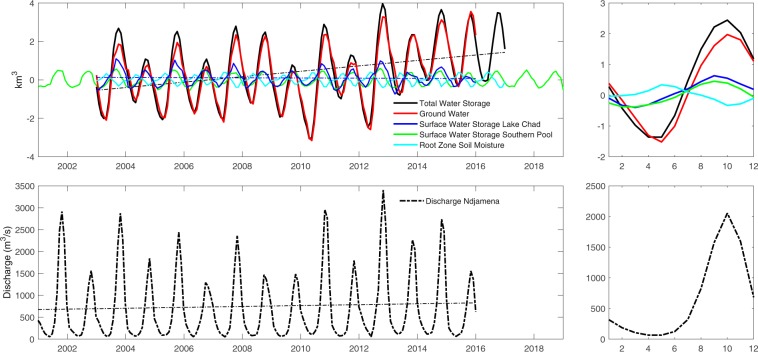
Top: monthly variation of GRACE-derived TWS (black), SWS of Lake Chad (blue) and its southern pool (green), groundwater (red), and root zone soil moisture (cyan). Bottom: monthly time series of *in situ* discharge at the N’Djamena gauge station.

Complementary satellite observations are used to estimate monthly variations of the surface and sub-surface water storage for the last 20 years (see Methods). When estimating the surface water storage (SWS) variations of Lake Chad and its southern pool, we observed that it exhibits no sensible trends over the last two decades, showing a significant seasonal variation with a mean annual amplitude of 1.2 km^3^ (Fig. 3 - top panel). Lake Chad’s SWS (blue curve) is dominated by the variation of water in the southern pool (green curve). SWS starts to increase slowly from March/April to July, before rising quickly to reach its maximum in September, then gradually decreases from October to its minimum in March. Monthly time series of *in situ* discharge at the N’Djamena gauge station (Fig. 3 - bottom panel) is compared to the variation of SWS. Although there is a 1-month time lag between the peak of discharge (October) and the peak of SWS (September), a high linear correlation (R = 0.86) is evidenced between these two independent time series considering the time lag. This time lag could be explained by the fact that the SWS in the southern pool reacts to rainfall that normally occur earlier than discharge of the Chari-Logone River.

The monthly variations of TWS anomalies from GRACE are used to estimate the relative contribution of sub-surface storages after removal of the SWS component. TWS exhibits a well-marked seasonal with an amplitude of 4 km^3^, similar to the one from SWS but more than 3 times higher. This implies that SWS only contributes to a maximum ~30% to the total variation of the water volume of the lake, and ~70% of water volume variations come from the subsurface. The water content of the subsurface can be separated in two components: the root zone soil moisture obtained from the Global Land Evaporation Amsterdam Model^25^ (see supporting document for details of the GLEAM dataset), and the groundwater after removal of the root zone soil moisture from the subsurface storage. We estimate that Lake Chad’s root zone soil moisture represents only 0.65 km^3^ of the total annual amplitude, meaning that TWS variations are mostly driven by the changes in groundwater which is in agreement with groundwater data observations from Sahel^26^. Lake Chad’s SWS and TWS exhibit similar time-variations (R = 0.81 over a 13-year period with one month of time lag). This delay could be attributed to the exchange of water from the surface reservoir to the subsurface/groundwater reservoirs, and by the slower subsurface/groundwater flow in comparison to the changes in surface water. Linear trends of SWS and TWS show slight increases of both surface water and total water volume for the 13-year and 14-year periods (0.002 km^3^ year^−1^ and 0.012 km^3^ year^−1^, respectively), with the maximum uncertainties on GW estimates is about 17% to 23% (see details in Methods). This increasing trend in the context of the current climate conditions confirms that the southern pool of the lake is not shrinking but, on the contrary, slightly increasing over the last 20 years as as an open lake modulated by the sill that separated from the northern pool. The southern pool is mainly supplied by the Chari-Logone River inputs that show increasing trends observed at four gauge stations along its catchment (Fig. 3 - bottom panel, and Fig. S11 in supporting information). Based on ^36^Cl concentration and stable oxygen data of both layers, it has been demonstrated that the deep aquifer water is composed of groundwater older than 1 Ma years, recharged during previous humid periods^27,28^. Because of the limited contribution of root zone soil moisture, the variation of TWS is strongly controlled by groundwater from the Quaternary Phreatic Aquifer. As recently studies estimated recharge rates of 78 ± 7 mm yr^−1^ in the lower Sahelian catchment, and of 240 ± 170 mm yr^−1^ in the upper humid sudanian catchment^21^, groundwater plays an important role in controlling the water cycle over the Lake Chad basin. When considering the porosity of the Lake Chad’s sediment is 0.1^21^, and the maximum depth of Lake Chad’s surface water is ~3 m (with a maximum contribution of 30% to the total variation of the water volume), we can estimate the depth of the Lake Chad’s groundwater aquifer is ~70 m which is consistent with the field observations^21^.

## Lake Chad in context of the climate change

**Figure 4:**
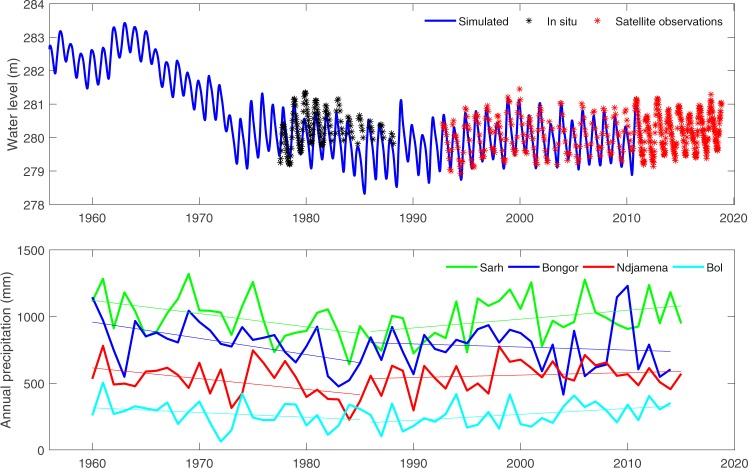
Top: comparison between *in situ* and satellite derived water height collected from Hydroweb^30^ (http://hydroweb.theia-land.fr), and simulated data over the southern pool of Lake Chad. Bottom: Annual precipitation at four gauge stations within the Lake Chad basin.

Combining ground-based observations^20^, modelling lake levels^19,29^ over the last seven decades, and our satellite-derived data over the last two decades, Lake Chad showed higher levels during the 1950s–60s compared to the present day (Fig. 4 - top panel). Despite the recovery of rainfall since mid 1980s (Fig. 4 - bottom panel), Lake Chad did not reach its level before the dry period of 1970s.

Several studies examined stream flow of Chari-Logone river and rainfall for the last 70 years as main indicators of the Lake Chad fluctuation^31,32^. They suggested that the lake is shrinking due to the decreasing trend observed in both parameters. A trend analysis from 1950 to 2018 highlights a decline in both streamflow of the main tributaries to the lake and rainfall as the level of current annual precipitation does not recover its amount observed during the 1950s. However, according to the variability of Sahelian rainfall and the consequent hydrological changes reflected by the lake, it is important to consider its changes at inter-annual time-scale. Figure 4 (bottom panel) clearly shows two sub-periods of rainfall over the Lake Chad basin, separated in mid 1980s. Similar trend is widely observed and defined in western and central Sahel^33–36^, attesting the annual rainfall increased again since the 1990s. Even if this increase in precipitation is at a smaller rate compared to what have been observed during the wettest decades 1950s–1960s, this period has been defined as a partial recovery of precipitation compared to the driest decades 1970s–1980s. For some authors^37^, the 1950s rainfall appears to be exceptional. But up to now, discussion on the trend of rainfall over the central Sahel at multidecadal scale remains an issue in spite of the availability of updated product as the CRU and compilation of in-situ measurements over the last 150 years, which confirmed that higher humid period compared to 1950s–1960s is not observed.

For now, the ongoing climatic changes is expected to enhance the global hydrological cycle under the Tropics and contributes to increase precipitation^38^. Several modeling experiments demonstrated that despite the important uncertainties to reproduce properly the tropical convection over the Sahelian area^39^, most of the global simulations are showing an increase in tropical rainfall, especially in the central Sahel^40^. When focused on the Chari-Logone catchment, this trend is confirmed with expected higher flow for the 21^*th*^ century^41^. This is in agreement to what we have learned from past reconstructions based on sedimentary archives collected in the Lake Chad basin. It was evidenced that during global cold phases, the lake is shrinking, whereas during global warm phases, the lake is rising^42,43^. During the last glacial maximum that occurred 20,000 years ago, Lake Chad was completely covered by an erg^44^. On the contrary, during the Holocene 10,000 years later, because of an earth-orbital configuration leading for higher isolation during summer months, Lake Chad reached its maximum extension as 25 times larger than today, with an area covering 350,000 km^2^ ^45^. However, some remote forcing factors could counterbalance the decadal trend. It has been evidenced that the sea surface conditions from North Atlantic ocean are closely linked with the rainfall in Sahel^34,46^, and the differential gradient between tropics and extratropics acts as a driver of rainfall through an atmospheric pathway which triggers the atmospheric circulation over the Sahel and Sahara^47^. Modeling experiments suggested that the freshwater discharge coming from Greenland melting could significantly impact the sea surface temperature of North Atlantic and induce a decrease in Sahel rainfall for the next decades^48^. Moreover, Sahelian rainfall is also affected by a higher interannual variability which is though to increase in the current climate change^5^. This trend has been evidenced for the whole Sahel, mainly due to mesoscale events that lead to extreme events and a variable amount of precipitation from year to year. Combined with the change of the rainy season length and the occurrence of the major amount of precipitation during rainy seasons^49^, this interannual variability is recognized for being one of the main factors affecting agriculture, livelihoods production and pastoral activities^50^.

In summary, our findings show that Lake Chad’s surface water extent slightly reduced over the last two decades, mostly in the northern pool due to the increase of evaporation and vegetation cover, as well as the decrease of the Komadugu Yobe discharge. However, the southern pool extent is stable and even, slightly increasing, as a consequence of stable local rainfall and the increase of the Chari-Logone river discharge. Despite the decrease in the northern pool, Lake Chad’s SWS shows an increasing trend for a 13-year period. Similar increasing trend is also observed in monthly variations of GRACE-derived TWS for the same period. The subsurface contributes to ~70% of Lake Chad’s TWS, meaning that most of its water is stored in soil moisture, and especially in groundwater with an estimated aquifer deep of ~70 m. As a consequence, despite of the uncertainties in predicting the future climate, for now Lake Chad is not disappearing. When considering the amount of water stored in the groundwater reservoir accessible by pumping, with the recharge rate in the active basin, it represents today one of the best opportunity for buffering the huge interannual variability in rainfall that characterizes the current climate changes in Sahel. This groundwater reservoir needs to be considered with attention for being integrated in appropriate development strategy and better managing the increasing pressure on resources in consequence of rapid population growth in Sahel.

## Methods

### Land surface water extent mapping with MODIS imagery

MODIS data have been used extensively in many studies for flood mapping and surface water extent monitoring at both regional^51–53^, as well as global scales^54^, by applying different criterions on several water indices. Among them, we chose to apply the methodology introduced in Berge-Nguyen *et al*.^53^ to extract surface water extent variation of Lake Chad because this method was specifically developed for the arid and semi-arid regions similar to the Lake Chad basin. A summary of the methodology is presented in Table 1. The terrain surface is classified into four classes, including open water (100% area of pixels is flooded (1)), mixed of water/dry land/aquatic vegetation (part of pixels is flooded, but the inunated ratio is unknown (2)), vegetation (3) and dry land (4). Note that cloud pixels (where values of the Visible Band 3 (blue; 459–479 nm) >= 0.2) are removed^51^, then all input bands are smoothed using the simple weight smoothing function, before being used for classification.

**Table 1:** Threshold values used for classifying terrain surface into four different classes using MODIS imagery^53^. Band 5 is the near-infrared (NIR) band (1230–1250 nm), and the Normalized Different Vegetation Index (NDVI) is calculated as the ration between the NIR Band 5 and the Visible Band 1 (red; 620–670 nm).

	Open Water	Mixed Water/Dry Land/	Vegetation	Dry Land
	(1)	Aquatic Vegetation (2)	(3)	(4)
Band 5	<=0.12	>0.12 & <=0.27	>0.27	>0.27
NDVI	No test	No test	>0.4	<=0.4

As the inundation ratio of mixed pixels is unknown, we estimate its optimal water ratio by comparing MODIS-derived surface water maps with the corresponding Landsat-derived surface water maps during a 6-year period (2013–2018), using Eq. (1):

1 $$\bar{R}=\frac{1}{k}\mathop{\sum }\limits_{i=1}^{k}\frac{\left(S{W}_{i}^{Landsat}-S{W}_{i}^{MODISwater}\right)}{{N}_{i}^{mixed}}$$

where SW_*i*_^*Landsat*^ and SW_*i*_^*MODISwater*^ are the corresponding surface water extent detected from Landsat-8 and MODIS water pixels, respectively. N_*i*_^*mixed*^ is the number of mixed pixels in the corresponding MODIS image, and $\bar{R}$ is the optimal water ratio for MODIS mixed pixels. After comparing 51 pairs of Landsat/MODIS imagery (see Fig. S1 in supporting information for their temporal distribution), we found that the optimal water ratio for MODIS mixed pixels is 18%. For the rest of the study, we will apply the water ratio of 18% for all MODIS mixed pixels (meaning that 18% of their area is covered by water). More details on validation of MODIS-derived surface water extent maps with Landsat-8 and Sentinel-2 products can be found in supporting information.

### Land surface water level mapping with satellite altimetry

Nowadays, satellite altimetry data are commonly used for land hydrology applications^55^, and it has been proved to be able to provide surface water level variation with good agreement compared to *in situ* data^56,57^ as long as the intersections between satellite ground-tracks and the water bodies are large enough. Over Lake Chad, six virtual stations (VSs) were defined at the cross-sections between ENVISAT/SARAL ground-tracks and the lake northern and southern pools and the surrounding floodplains (Fig. 1b). There are three VSs (1, 2, and 4) located along the Archipelagos, one VS (3) located in the southern pool, one VS (6) located in the northern pool, and one VS (5) located near the Great Barrier. Time series of water levels based on the ICE-1 retracked ranges which provides accurate estimate of the height of the lake water bodies^56^, were derived using the Multi-mission Altimetry Processing Software (MAPS), that is commonly used for this purpose^58,59^. Details of the processing of the altimetry data in MAPS can be found in Normandin *et al*.^60^. As already mentioned, only altimetry data from the 35-day repeat period missions are used, meaning that there are 11 values of water level per year per VS. Although altimetry data provide instantaneous measurements over the area, we assume that water dynamics of Lake Chad is smooth enough to make results derived from instantaneous measurements are not far from the monthly mean values of the lake^61^. Values of the missing months are filled using the simple weight smoothing function to construct monthly time series of surface water level of Lake Chad for the 2003–2018 period. For the southern pool however, the water height is also extracted from Hydroweb which gives a long time series (from 1992 until mid 2019), including also data from Topex/Poseidon, Jason-1, Jason-2 and Jason-3 benefiting from higher temporal spaning of 10 days^30^. It has been used to fill the gap of the VSs between 2010 and 2013 by applying a modulation function between water level of all VSs and the water level from Hydroweb over the Lake Chad’s southern pool

Monthly surface water level maps of Lake Chad were estimated combining radar altimetry-based surface water levels at six VSs and MODIS-derived surface water extent maps. Every month, satellite-derived water levels at all six VSs are linearly interpolated over the inundated pixels detected in the corresponding MODIS-derived surface water extent map to estimate the surface water level at each grid point of 500 m MODIS spatial resolution following the approach developed by Frappart *et al*.^62^. This approach was also applied successfully in other basins worldwide^63–65^. Then, we successfully constructed monthly surface water level maps of Lake Chad at 500 m spatial resolution for the 2003–2015 period. Examples of surface water level maps of Lake Chad can be found in Fig. S10 in supporting information.

### Water volume variations

Monthly surface water volume variation of Lake Chad corresponds to the difference of surface water levels integrated over the monthly MODIS-derived inundated areas. The variation *δ*V(t_*i*_, t_*i*−1_), between two consecutive months numbered *i* and *i−1*, over floodplain *S*, is estimated using the following equation^66^:

2 $$\delta V(i,i-1)=\frac{({H}_{i}-{H}_{i-1})\times \left({S}_{i}+{S}_{i-1}+\sqrt{{S}_{i}\times {S}_{i-1}}\right)}{3}$$

where *δ*V represents the volume variation between two consecutive measurements, H_*i*_, H_*i*−1_ and S_*i*_, S_*i*−1_ are the corresponding maps at 500 m spatial resolution of surface water height and surface water extent of Lake Chad for months *i* and *i−1*, respectively. The unit of the surface water volume variation is expressed in km^3^. For floodplain area S, we take 100% for water pixels, and 18% for mixed pixels.

The variation total water storage (TWS) of Lake Chad is the sum of SWS, groundwater and root zone soil moisture (called subsurface water storage; SSWS). The spatial temporal variation of TWS and root zone soil moisture are estimated using GRACE and GLEAM dataset, respectively, and the variation of SWS is estimated using Eq. (2)^64,67^. Therefore, monthly variation of groundwater can be calculated as the difference between variations of TWS, and root zone soil moisture and SWS (Eq. (3)):

3 $$\Delta TWS=\Delta SWS+\Delta SoilMoisture+\Delta Groundwater$$

Variations of TWS, SWS, root zone soil moisture and groundwater are all expressed in km^3^.

### Uncertainties of this study

The decomposition technique employed in the present study to estimate groundwater is commonly used (see the review from Frappart & Ramillien^64^ on GRACE and groundwater for instance), and several specific regional studies proved that it is a powerful tool^68–70^.

Following Frappart *et al*.^69^, the uncertainty on groundwater storage variations can be computed as follows:

4 $${\sigma }_{GW}=\sqrt{{({\sigma }_{TWS})}^{2}+{({\sigma }_{SWS})}^{2}+{({\sigma }_{SM})}^{2}}$$

Based on Ramillien *et al*.^71^, we assumed that *σ*_*TWS*_ < 0.015 m over semi-arid area.

Based on Frappart *et al*.^68^ and Papa *et al*.^70^, we assumed that: *σ*_*SWS*_ < max(△*h*)*σ*_*Sf lood*_ + max(*S*_*f lood*_)*σ*_*h*_

Over Chad Lake, the maximum change of water level from one month to another (max(△*h*)) is 0.15 m, the maximum surface of the lake during the observation period (max(△*Sf lood*)) is 16,800 km^2^, the associated maximum error (*σ*_*Sf lood*_) is 8.5 of this latter value, the maximum error on the water level is assumed to 0.03 m (see Birkett, 2000^14^). Over low vegetation area, GLEAM soil moisture estimates were found to perform well when compared to in-situ data^72^. Over semi-arid areas in Australia for instance, a maximum error of 2.6% was found. We assumed this value remains in the same order of magnitude over Lake Chad region.

As a result, when taking into account all those maximum values of uncertainties, we estimate that:

max(*σ*_*GW*_) = 0.7 km^3^ for an annual amplitude between 3 and 4 km^3^ at the grid point scale. max(*σ*_*GW*_) = 7.5 km^3^ for an annual amplitude between 55 and 65 km^3^ over the whole Lake Chad Quaternary aquifer (Fig. 1a).

We conclude that the maximum uncertainties on GW estimates in our study is about 17% to 23%.

## Supplementary information

Supplementary Information.
