# Supplementary material for: The Lake Chad hydrology under current climate change
Source: Sci Rep. 2020 Mar 26;10:5498. doi: 10.1038/s41598-020-62417-w (PMC7099084; doi:10.1038/s41598-020-62417-w)
Supplement: Supplementary file 1 — Supplementary Information. [file 41598_2020_62417_MOESM1_ESM.pdf]

Supporting information for "The Lake Chad hydrology under current climate change"

Binh Pham-Duc[1,2,*], Florence Sylvestre[1], Fabrice Papa[3,4], Frédéric Frappart[3], Camille Bouchez[5], and Jean-Francois Crétaux[3]

[1]*Aix-Marseille Université, CNRS, IRD, Collège de France, INRA, CEREGE, Europôle de l'Arbois, Aix-en-Provence, France*

[2]*Department of Space and Applications, University of Science and Technology of Hanoi, Vietnam Academy of Science and Technology, Hanoi, Vietnam*

[3]*LEGOS, Université de Toulouse, IRD, CNES, CNRS, UPS, Toulouse, France*

[4]*Universidade de Brasília, Institute of Geosciences, Campus Universitario Darcy Ribeiro, 70910-900 Brasilia (DF), Brazil*

[5]*Université Rennes, CNRS, Géosciences Rennes, UMR 6118, Rennes, France*

Datasets

We use MODIS/Terra atmospherically corrected surface reflectance 8-day Level 3 Global 500 m SIN Grid V006 imagery (MOD09A1) for detecting and monitoring monthly variation of surface water extent of Lake Chad at 500 m spatial resolution, for the 2001-2018 period. Although MODIS has moderate spatial resolution compared to other satellite products (for example, Landsat at 30 m and Sentinel-2 at 10-20 m). However, MODIS presents a strong advantage with its temporal resolution since it offers 2 images per day compared to 16 days for Landsat or 10 days with Sentinel-2. Moreover, it offers longer time series than Sentinel-2 which observations started in mid-2015. MOD09A1 imagery used in this study are freely distributed from the NASA's Earth-Data Hub (`https://search.earthdata.nasa.gov/search`). For validation purposes with MODIS surface water maps, we use atmospherically corrected surface reflectance Lansat-8 and Sentinel-2 imagery at 30 m and 10 m spatial resolution, respectively. Landsat-8 imagery are ordered from the USGS EarthExplorer website (`https://earthexplorer.usgs.gov/`), and Sentinel-2 Level-1C Top-of-Atmosphere (TOA) imagery are downloaded from the Sentinel Data Hub (`https://scihub.copernicus.eu/dhus/#/home`). To limit the effects of clouds, only Landsat and Sentinel-2 imagery with <= 5% of cloud contamination are used. Temporal distribution of all 51 Landsat-8 and 43 Sentinel-2 imagery used in this study is shown in Figure S1.

Radar satellite altimetry data from two 35-day repeat period missions (ENVISAT, and SARAL) are used to estimate monthly variation of surface water levels at different parts of Lake Chad for

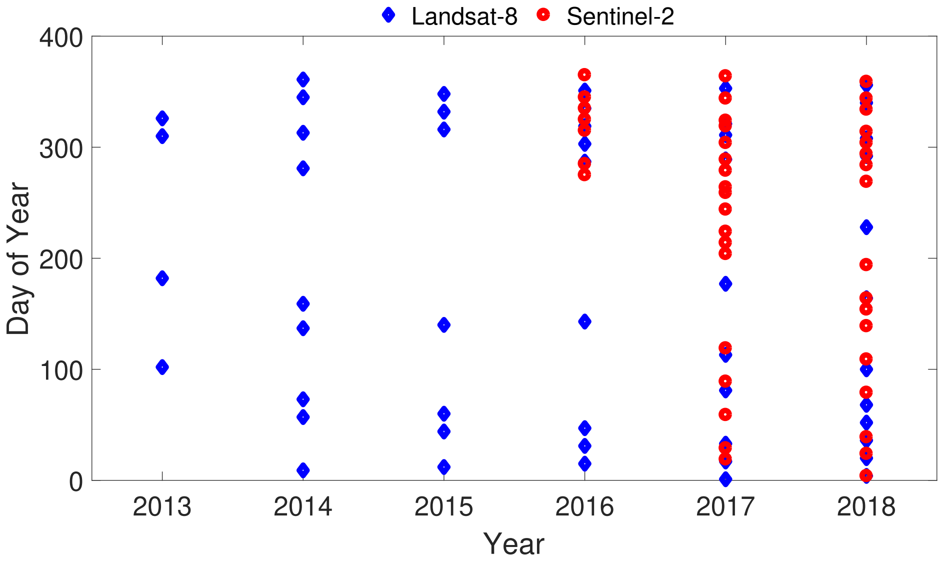

Figure S1: Temporal distribution of 51 Landsat-8 and 43 Sentinel-2 imagery used in this study.

the 2003-2015 period. All altimetry data used in this study were processed, validated, and distributed by the Center of Topography of the Oceans and the Hydrosphere (CTOH) in the Laboratoire d'Études en Géophysique et Océanographie Spatiales (LEGOS), France (`http://ctoh.legos.obs-mop.fr/`). Note that altimetry data from missions with a 10-day repeat period (for instance, Jason-1,2,3) are available but only used to construct the water height of the southern pool as there is no intersection between the satellite ground-tracks and other parts of Lake Chad. For the southern pool, we use directly water height data collected from the Hydroweb[1] (`http://hydroweb.theia-land.fr/`).

GRACE Land Mass Grids - Global mascons products (Release 06) (`https://grace.jpl.nasa.gov/data/get-data/`) are also used to estimate monthly variation of total land surface water storage for the 2003-2016 period, with an accuracy of ∼1.5 cm of equivalent water thickness[2,3]. Monthly GRACE data are provided by three different processing centers: the Geo-

forschungsZentrum Potsdam (GFZ), the Center for Space Research at University of Texas, Austin (CSR), and the Jet Propulsion Laboratory (JPL). The three products are averaged to reduce noise in the gravity field solutions[4], then the output is multiplied with the provided GRACE scaling factor to increase the accuracy of the GRACE total water storage estimates[5]. Although GRACE spatial resolution is ∼300 km, the product we use is distributed on a 0.25°×0.25° pixel-size grid.

We use GLEAM 3.3 version dataset[6,7] to estimate monthly variation of root-zone soil moisture over Lake Chad at 0.25°×0.25° pixel-size grid, for the 2003-2016 period. The dataset is distributed at `https://www.gleam.eu/` where several details can be found.

Figure S2 shows all satellite datasets used in this study, and some available satellite datasets could be used in future studies.

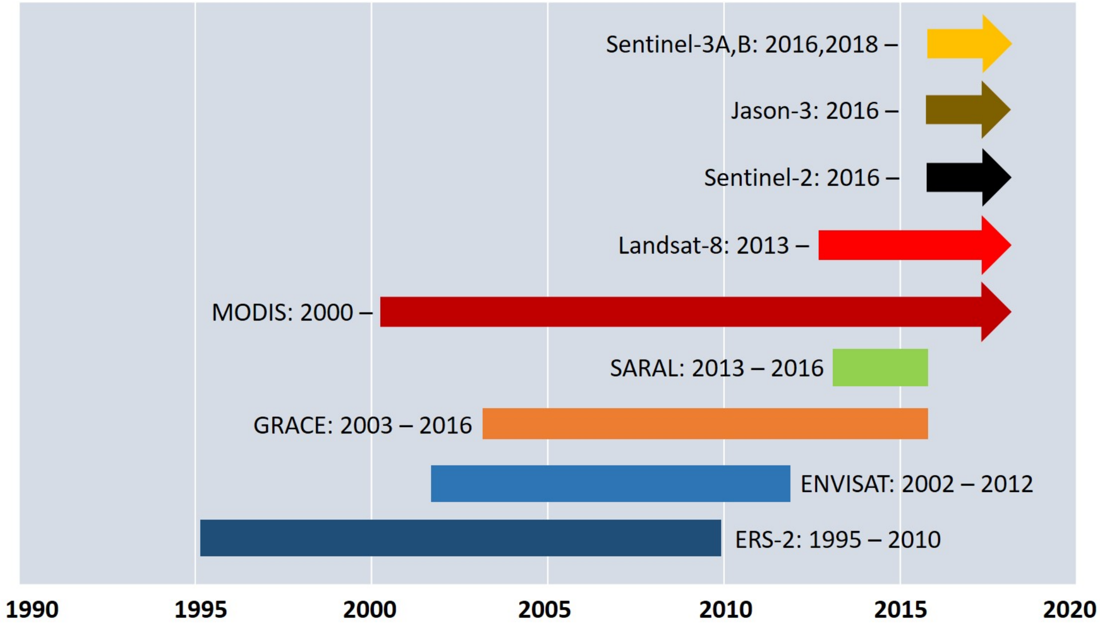

Figure S2: List of satellite datasets used in this study.

Land Surface Water Extent Mapping with MODIS Imagegy

Examples of monthly surface water extent maps derived from MODIS imagery over Lake Chad for January, April, July and October 2003 is shown in Figure S3. Vegetation cover over the Lake is also presented. It is clear that vegetation cover is limited at the beginning of the year (in January), then it increases gradually in April and July until it reaches the maximum when the wet season comes in October.

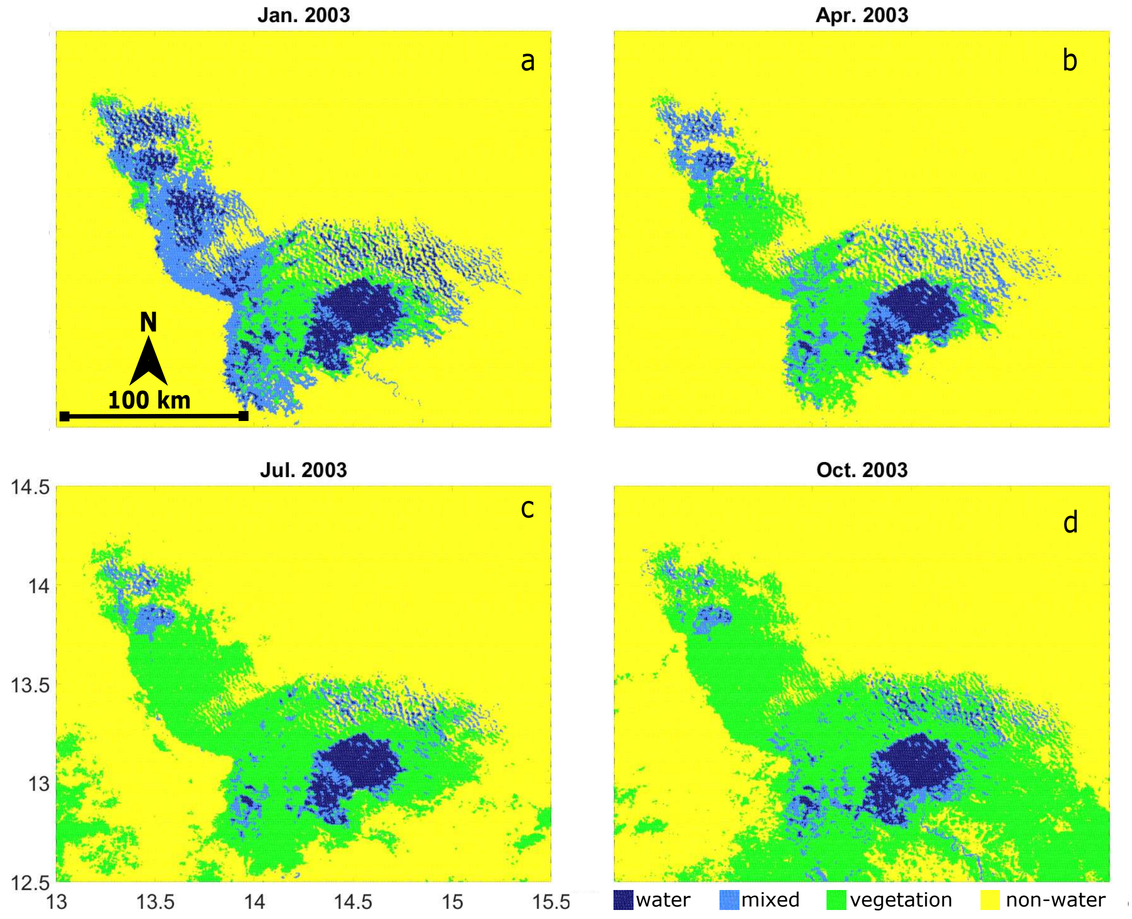

Figure S3: Examples of surface water extent maps derived from MODIS (at 500 m spatial resolution) over Lake Chad for January, April, July and October 2003

Land Surface Water Extent Mapping with Landsat-8 and Sentinel-2 Imagery.

The variations of surface water extent at both regional and global scales have been monitored using higher spatial resolution images from Landsat[8–11], and recently Sentinel-2[12,13] observations. The main principle of these studies is to distinguish water and non-water bodies based on the application of water indices[13], such as the Normalized Difference Vegetation Index (NDVI)[14], the Normalized Difference Water Index (NDWI)[15], the Modified Normalized Difference Water Index (MNDWI)[16], or the Automated Water Extraction Index (AWEI)[17]. Some authors compared the performance of these indices in different regions[12,16,18–20], and concluded that the MNDWI normally gives the best result among all the indices. Over Lake Chad basin, the MNDWI was also reported to work better than other indices in detecting surface water bodies[20,21]. In this study, we apply a threshold on the MNDWI to separate water and non-water bodies within Lake Chad region. By definition, the MNDWI is the ratio between the green band and the middle infrared band[16] (see Equation (1)).

$$MNDWI = \frac{Green - MIR}{Green + MIR} \tag{1}$$

Over water bodies, MIR wavelengths (1550-1750 nm) are almost completely absorbed and the green wavelengths (520-600 nm) are highly reflected. As a consequence, water surfaces usually have high positive MNDWI values. In contrast, vegetation canopy and soil usually have low negative MNDWI values because the MIR wavelengths are reflected more than the green wavelengths. A threshold (T = 0) is normally set in order to distinguish water from non-water pixels. However, we found that with this threshold, the derived surface water extent maps within Lake Chad are overestimated, especially over the north part of the southern pool. Some authors applied

Otsu algorithm[22] to automatically extract surface water bodies as it is one of the best thresholding techniques[20]. To obtain the best result, the Otsu algorithm requires that the image to be classified must have bi-modal or multi-modal histogram distribution. This method does not provide the best result if the histogram is uni-modal or close to uni-modal. We tested the Otsu algorithm with both Landsat-8 and Sentinel-2 images, but results were not satisfied because the MNDWI histogram distributions were not bi-modal as the number of non-water pixels is much larger than the number of of water pixel (see Figure S4). After many careful tests on different thresholds between 0 and the Otsu threshold (0.3176), the MNDWI threshold for Lake Chad was set to 0.2. This is consistence with Zhu et al. (2017)[21] which also applied the same threshold value for classifying surface water extent over the southern pool of Lake Chad. Table S1 shows the corresponding green and MIR bands from Landsat-8 and Sentinel-2 used to calculate the MNDWI. For Sentinel-2 imagery, the spatial resolutions of green and MIR bands are 10 m and 20 m, respectively. To produce MNDWI at 10 m spatial resolution, the MIR band is resampled to downscale the spatial resolution from 20 m to 10 m using image fusion technique (e.g., pan-sharpening[23]). All Landsat-8 and Sentinel-2 pre-processing steps are processed using ENVI and ESA's SNAP software. See the temporal distribution of Landsat-8 and Sentinel-2 used in this study in Figure S1.

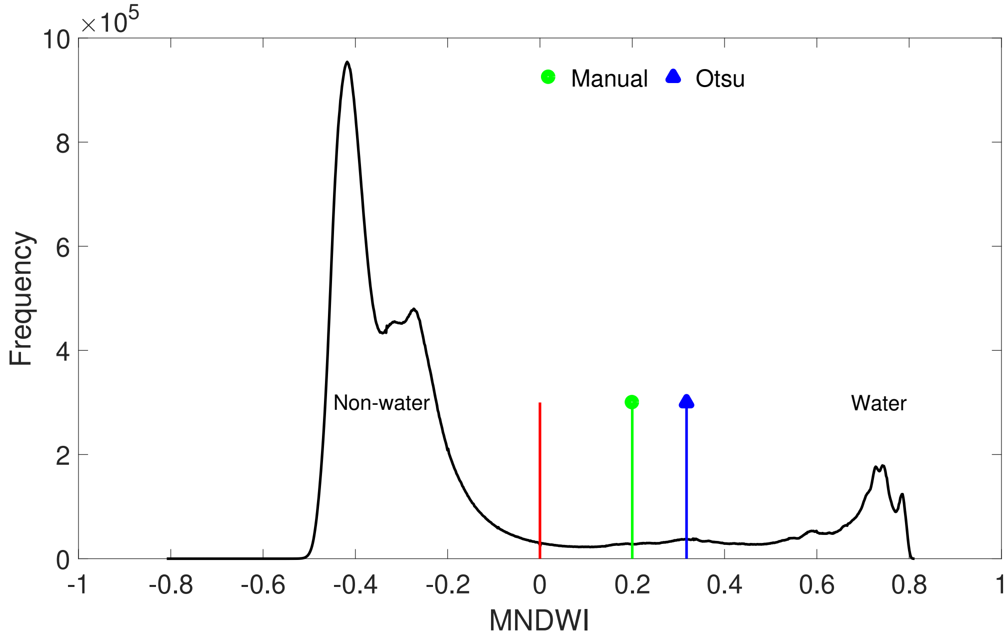

Figure S4: Histogram of a Sentinel-2 MNDWI imagery over Lake Chad (acquired on 6 October 2017) with three different threshold values.

Table S1: Corresponding Landsat-8 and Sentinel-2 Green and MIR bands used to calculate the MNDWI.

	**Green band**	**MIR band**
**Landsat-8**	Band 3 (530-590 nm; 30 m)	Band 6 (1570-1650 nm; 30 m)
**Sentinel-2**	Band 3 (523-595 nm; 10 m)	Band 11 (1520-1700 nm; 20 m)

Validation of MODIS-derived Surface Water Extent Maps

To validate our MODIS-derived surface water extent maps, we compared with results derived from Landsat and Sentinel-2 imagery. Figure S5 shows inundation frequency of Lake Chad derived from 18 years (2001-2018) of MODIS data (left), and from 32 years (1984-2015) of Landsat data (right)[11]. The structure of Lake Chad inundation frequency is very similar, with high inundated ratio in the southern pool, lower inundated ratio in the Archipelagos, and much lower in the northern pool, as expected. Between the two pools, there is a non-inundated area as this part is always covered by permanent vegetation. At this stage, we are not sure that this area is totally dry, or there are water under the vegetation canopy but they are invisible from satellite sensors (both optical and radar). Results from Landsat data suggest that the inundated ratio in the northern pool and in the Archipelagos is higher than from MODIS. This can be explained by the fact the the time

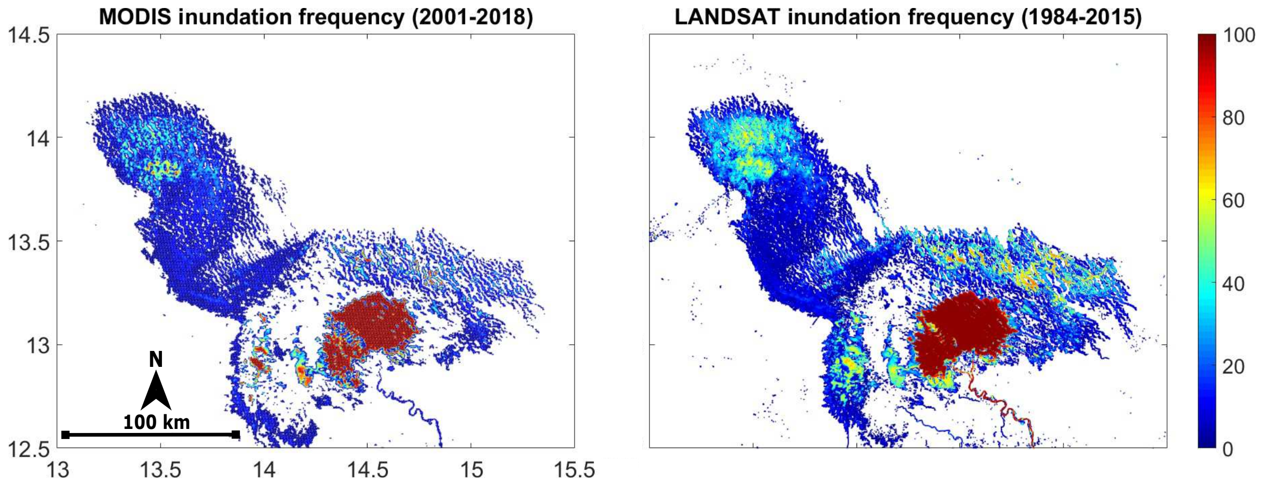

Figure S5: Inundation frequency of Lake Chad derived from 18 years of MODIS (left) and 32 years of Landsat data (right).

series of Landsat data used in Pekel et al. (2016)[11] for the estimation is nearly double compared to the time series of MODIS data we used in this study (32 years compared to 18 years). Permanent surface water extent (when the surface are inundated >= 80% during the study period) derived from MODIS and Landsat are 1595 km^2 and 1515 km^2, respectively. As Landsat have higher spatial resolution than MODIS, Landsat image shows many details of small water bodies than MODIS (for example, the tiny river in the southwest of the southern pool).

Very good agreement are also evidenced when compare MODIS-derived with Sentinel-2-derived surface water extent maps of the southern pool in dry and wet seasons 2017 (Figure S6). The southern pool appears clearly in both MODIS and Sentinel-2 surface water extent maps, and advantages of higher Sentinel-2 spatial resolution (10 m) are obviously seen. Rivers and small water bodies in the Archipelagos are totally detected in Sentinel-2 maps, but partly or not detected in MODIS maps. 43 free-cloud Sentinel-2 imagery (<= 5% cloud contamination) are processed to obtain the time series of surface water extent in the southern pool for the October 2016 - December 2018 period, for comparison with MODIS results (Figure S7). The two time series show a very high linear temporal correlation (96%), but MODIS always underestimates surface water extent compared to Sentinel-2. This is expected because the spatial resolution of Sentinel-2 is 250 times higher than MODIS, therefore, Sentinel-2 can detect much better small water bodies than MODIS. Total surface water area detected by Sentinel-2 is 14%-15% higher than that detected by MODIS. The difference in the rainy seasons is lower (9%-10%) than in the dry seasons (18%-20%). The difference mostly comes from the river, and especially from the Archipelagos where the environment is very complicated by the combination of soil, water and sand.

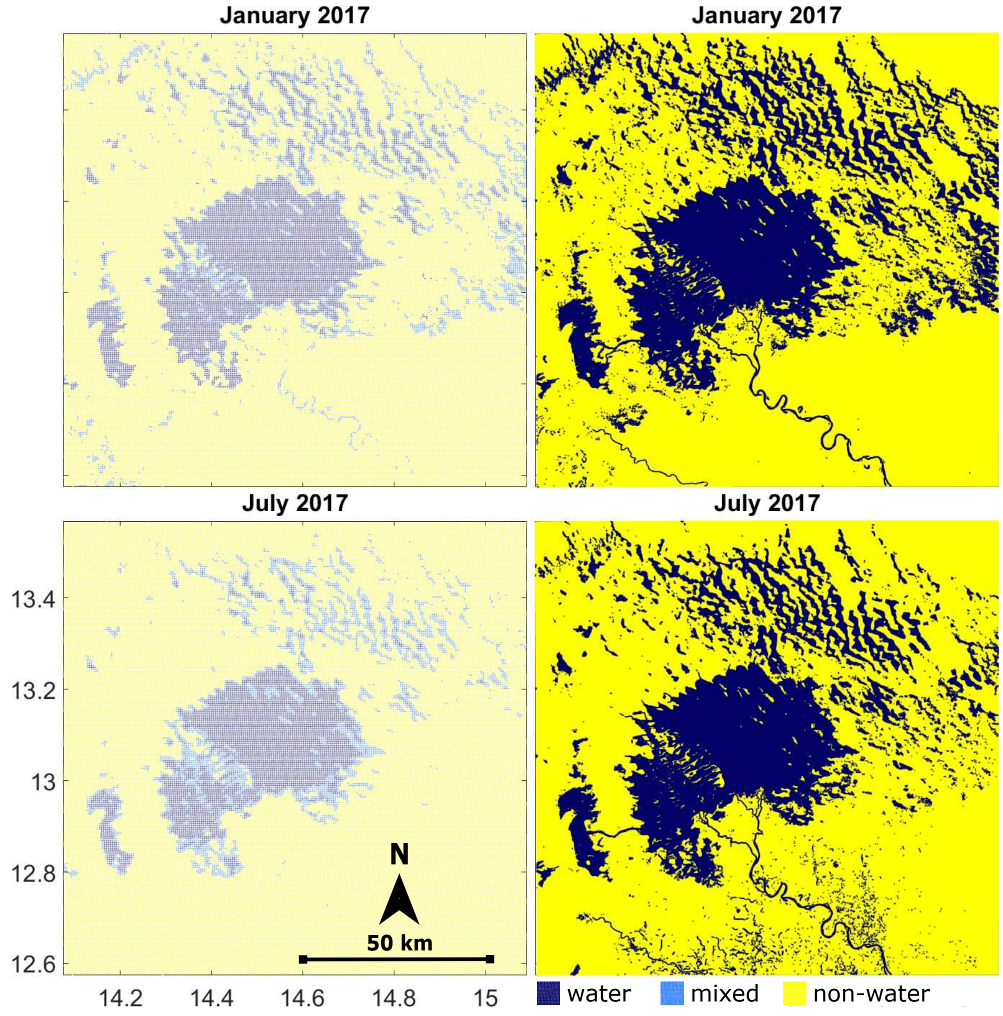

Figure S6: Surface water extent maps over the southern pool of Lake Chad, derived from MODIS (left) and Sentinel-2 (right) in dry and rainy seasons 2017. Sentinel-2 images were acquired on 29 January and 27 July 2017, respectively.

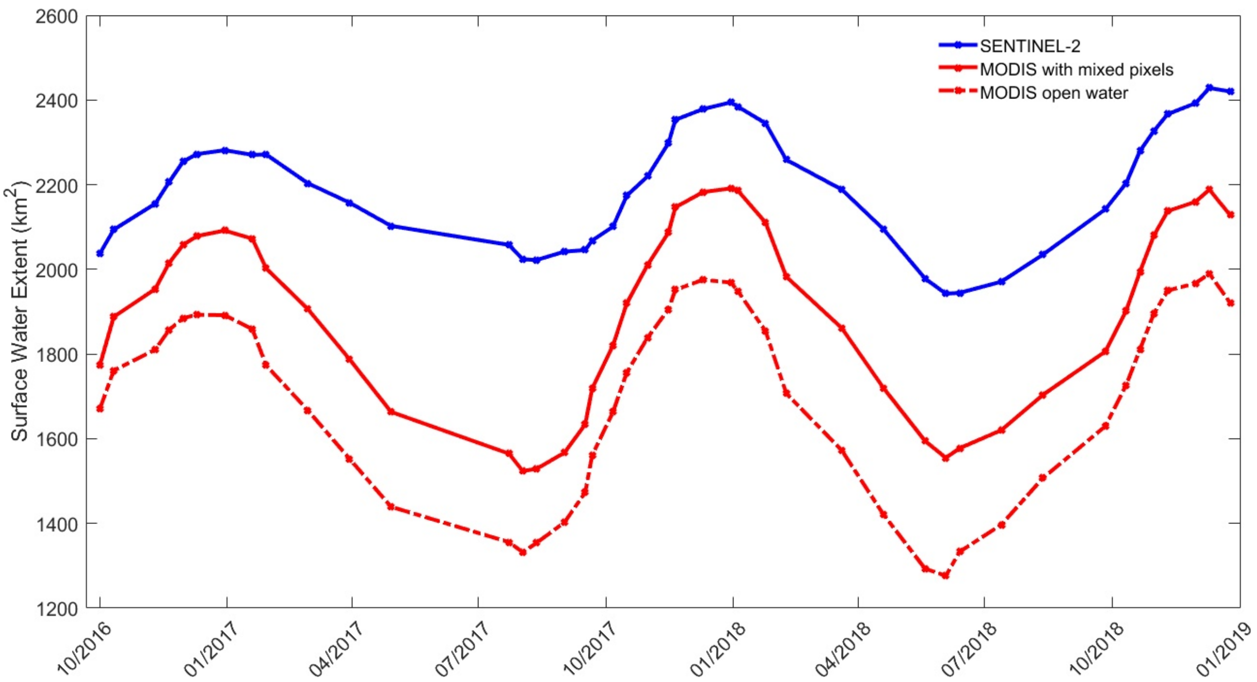

Figure S7: Comparison between time series of surface water extent over the southern pool of Lake Chad derived from MODIS and Sentinel-2 for the October 2016 - December 2018 period. See Figure S1 for the temporal distribution of Sentinel-2 images.

Annual variation of surface water and permanent vegetation

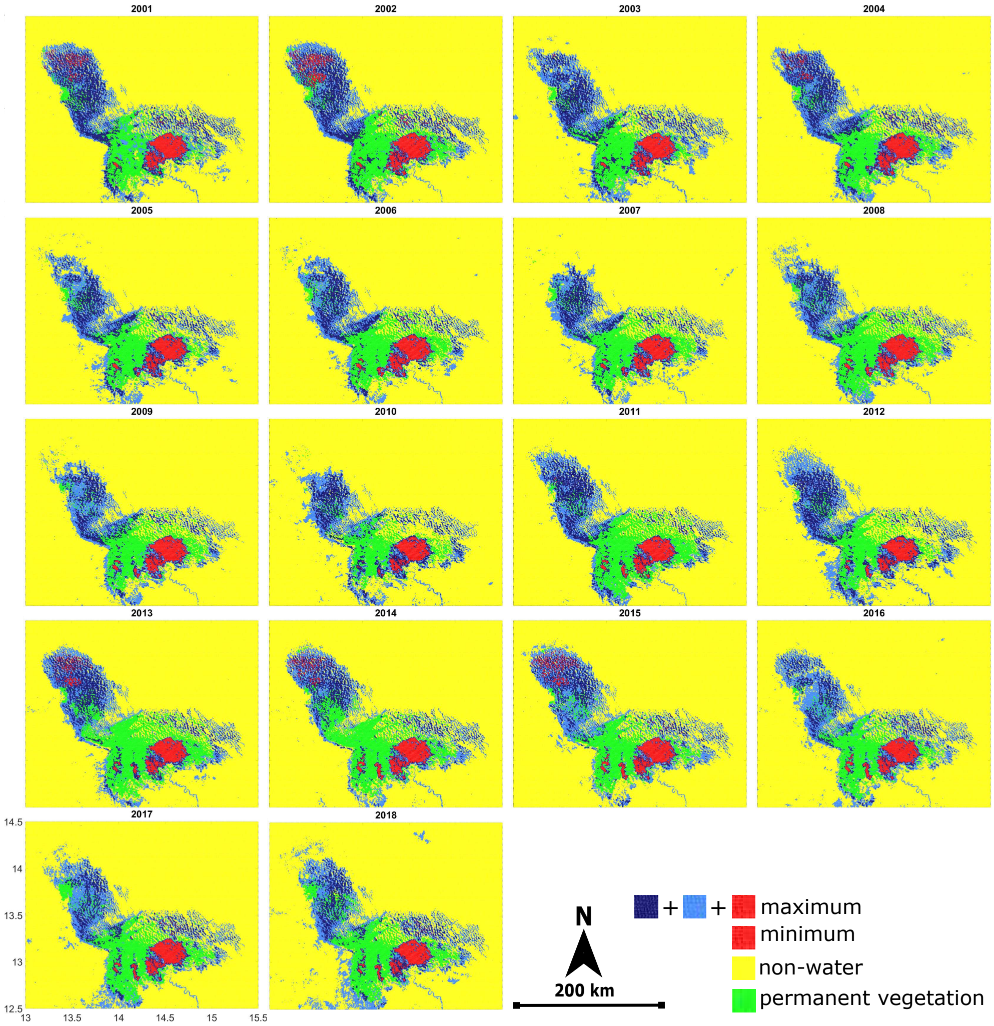

Figure S8: Annual minimum (red) and maximum (red, light and dark blue) surface water extent, and permanent vegetation (green) of Lake Chad from 2001 to 2018.

Annual minimum (red) and maximum (red, light and dark blue) of surface water extent of Lake Chad over the last 18 years are shown in Figure S8. It clearly shows that the southern pool of Lake Chad is very stable during almost the last 20 years, at both minimum and maximum states. In the northern pool, surface water extent variation is more dynamic.

Figure S9 shows the increase of permanent vegetation cover between the two pools as shown in Figure S8. An increase of ∼30% of permanent vegetation is evidenced between 2001 (∼3800 km^2) and 2018 (∼5200 km^2).

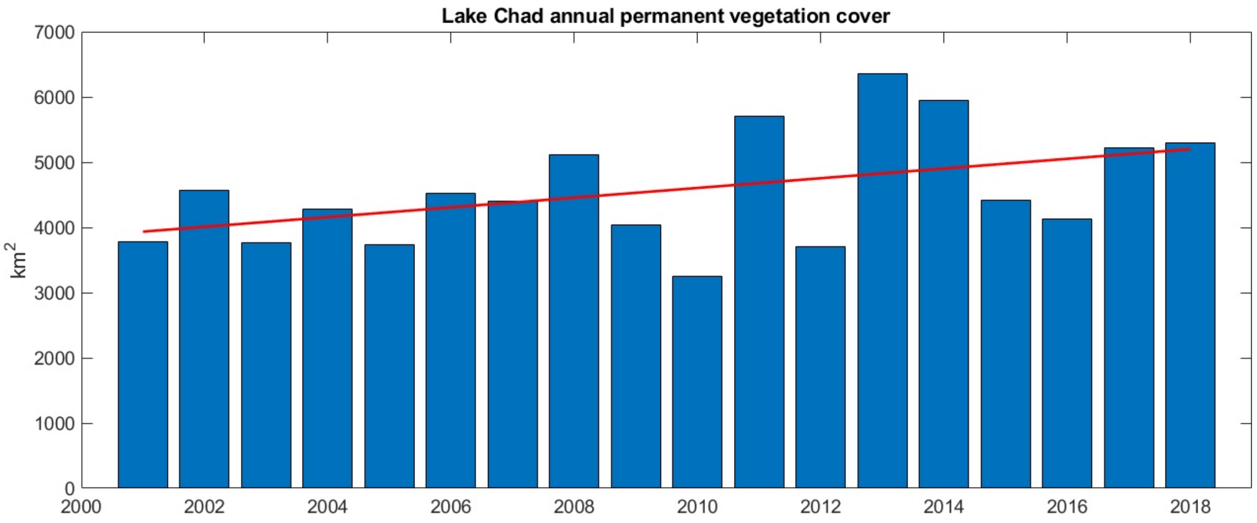

Figure S9: Annual permanent vegetation cover over Lake Chad for the 2001-2018 period.

Monthly surface water level maps

Examples of monthly surface water level maps over Lake Chad in January, April, July, and October 2003 are shown in Figure S10.

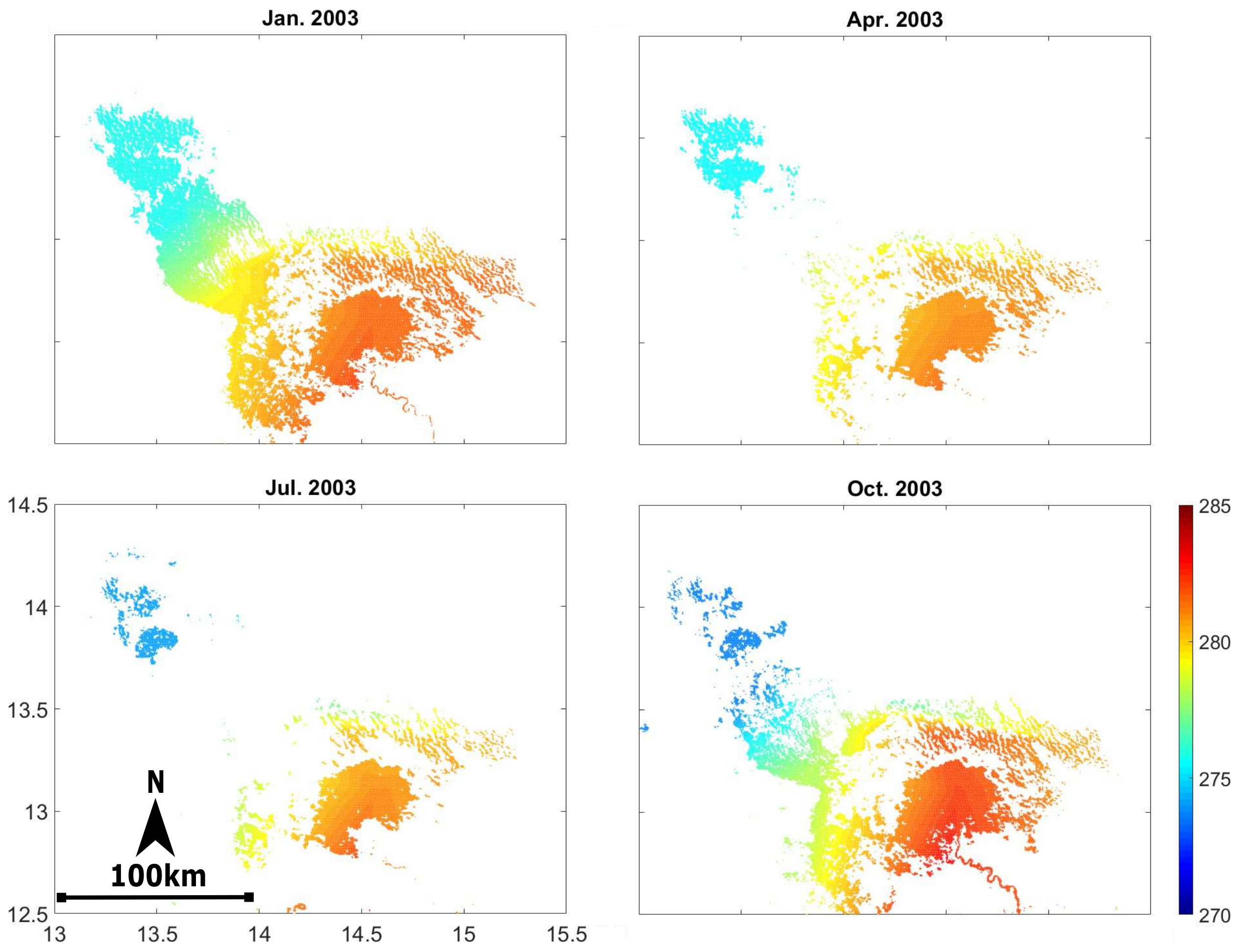

Figure S10: Surface water level maps (in m) at 500 m spatial resolution for January, April, July, and October 2003.

Variation of river discharge

Monthly in situ discharge data at four gauge stations, for the 1985-2015 period, are collected and shown in Figure S11. The longest time series data (back to 1950) are only available in N'Djamena station. Figure S11 clearly shows the decreasing trend of discharge from 1950s to the end of 1980s, but from the beginning of 1990s until present time, discharge is slowly increasing again.

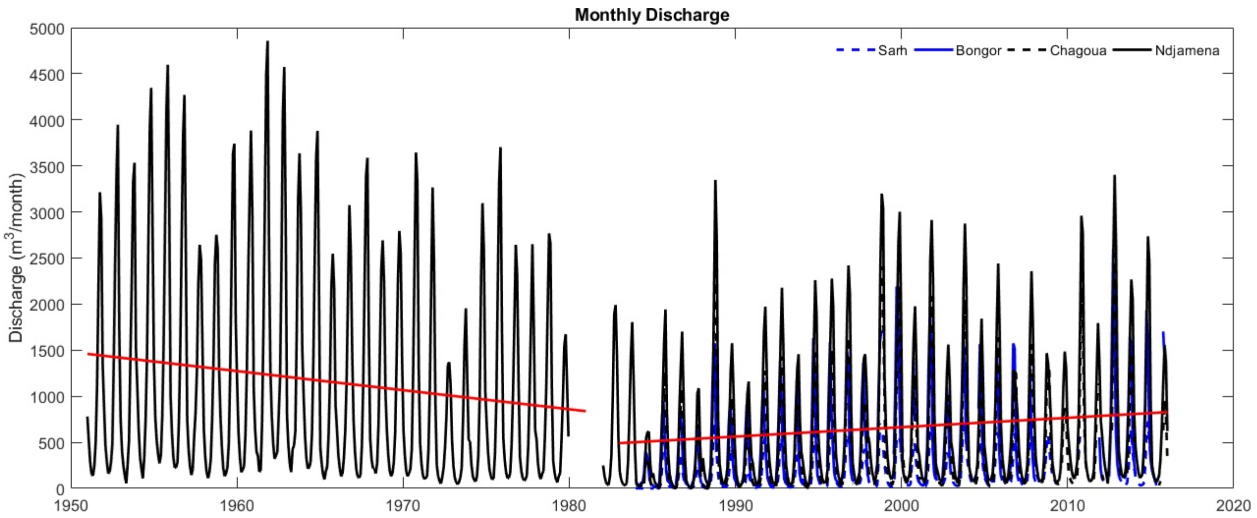

Figure S11: Monthly in situ discharge time series at four gauge stations (N'Djamena, Chagoue, Bongor, and Sarh). Their trends (red) are also plotted.

1. Cretaux, J.-F. *et al.* SOLS: A lake database to monitor in the Near Real Time water level and storage variations from remote sensing data. *Advances in Space Research* **47**, 1497 – 1507 (2011).

2. Ramillien, G., Frappart, F., Cazenave, A. & Guntner, A. Time variations of land water storage from an inversion of 2 years of GRACE geoids. *Earth and Planetary Science Letters* **235**, 283 – 301 (2005).

3. Frappart, F. *et al.* Remote Sensing of Environment Satellite-based estimates of groundwater storage variations in large drainage basins with extensive floodplains. *Remote Sensing of Environment* **115**, 1588–1594 (2011).

4. Sakumura, C., Bettadpur, S. & Bruinsma, S. Ensemble prediction and intercomparison analysis of GRACE time-variable gravity field models. *Geophysical Research Letters* **41**, 1389–1397 (2014).

5. Landerer, F. W. & Swenson, S. C. Accuracy of scaled GRACE terrestrial water storage estimates. *Water Resources Research* **48** (2012).

6. Miralles, D. G. *et al.* Global land-surface evaporation estimated from satellite-based observations. *Hydrology and Earth System Sciences* **15**, 453–469 (2011).

7. Martens, B. *et al.* GLEAM v3: satellite-based land evaporation and root-zone soil moisture. *Geoscientific Model Development* **10**, 1903–1925 (2017).

8. Du, Z., Li, W., Zhou, D., Tian, L. & Ling, F. Analysis of Landsat-8 OLI imagery for land surface water mapping. *Remote Sensing Letter* **5**, 672–681 (2014).

9. Mueller, N. *et al.* Water observations from space: Mapping surface water from 25 years of Landsat imagery across Australia. *Remote Sensing of Environment* **174**, 341–352 (2016).

10. Yamazaki, D., Trigg, M. A. & Ikeshima, D. Development of a global 90m water body map using multi-temporal Landsat images. *Remote Sensing of Environment* **171**, 337–351 (2015).

11. Pekel, J.-F., Cottam, A., Gorelick, N. & Belward, A. S. High-resolution mapping of global surface water and its long-term changes. *Nature* **540**, 418–422 (2016).

12. Du, Y. *et al.* Water Bodies ' Mapping from Sentinel-2 Imagery with Modified Normalized Difference Water Index at 10-m Spatial Resolution Produced by Sharpening the SWIR Band. *Remote Sensing* **8** (2016).

13. Yang, X., Zhao, S., Qin, X., Zhao, N. & Liang, L. Mapping of Urban Surface Water Bodies from Sentinel-2 MSI Imagery at 10 m Resolution via NDWI-Based Image Sharpening. *Remote Sensing* **9** (2017).

14. Rouse Jr., J., Haas, R., Schell, J. & Deering, D. Monitoring Vegetation Systems in the Great Plains with ERTS. *NASA Special Publication* **351**, 309 (1974).

15. McFeeters, S. K. The use of the Normalized Difference Water Index (NDWI) in the delineation of open water features. *International Journal of Remote Sensing* **17**, 1425–1432 (1996).

16. Xu, H. Modification of Normalised Difference Water Index (NDWI) to enhance open water features in remotely sensed imagery. *International Journal of Remote Sensing* **27**, 3025–3033 (2006).

17. Feyisa, G. L., Meilby, H., Fensholt, R. & Proud, S. R. Automated Water Extraction Index : A New Technique for Surface Water Mapping Using Landsat Imagery. *Remote Sensing of Environment* **140**, 23–35 (2014).

18. Li, W. *et al.* A Comparison of Land Surface Water Mapping Using the Normalized Difference Water Index from TM, ETM+ and ALI. *Remote Sensing* **5**, 5530–5549 (2013).

19. Singh, K. V., Setia, R., Sahoo, S., Prasad, A. & Pateriya, B. Evaluation of NDWI and MNDWI for assessment of waterlogging by integrating digital elevation model and groundwater level. *Geocarto International* **30**, 650–661 (2015).

20. Buma, W. G., Lee, S. I. & Seo, J. Y. Recent surface water extent of Lake Chad from multi-spectral sensors and GRACE. *Sensors* **18** (2018).

21. Zhu, W., Yan, J. & Jia, S. Monitoring Recent Fluctuations of the Southern Pool of Lake Chad Using Multiple Remote Sensing Data: Implications for Water Balance Analysis. *Remote Sensing* **9** (2017).

22. Otsu, N. A threshold selection method from gray-level histograms. *IEEE Transactions on Systems, Man, and Cybernetics* **9**, 62–66 (1979).

23. Vivone, G. *et al.* A critical comparison among pansharpening algorithms. *IEEE Transactions on Geoscience and Remote Sensing* **53**, 2565–2586 (2015).
